# Multi-omics profiling and cellular validation reveal distinct lipid networks of krill oil and its predicted mechanisms against insulin resistance

**DOI:** 10.3389/fnut.2026.1925480

**Published:** 2026-09-23

**Authors:** Jinxin Lin, Jinfeng Zhou, Shiyu Luo, Haocheng Liu, Jiguo Yang

**Affiliations:** 1 Medical Education Center of Jinan University/Department of Endocrinology, Guangdong Provincial People’s Hospital (Guangdong Academy of Medical Sciences), Southern Medical University, Guangzhou, China; 2 School of Food Science and Engineering, South China University of Technology, Guangzhou, China; 3 School of Public Health and Health Management, Gannan Medical University, Ganzhou, Jiangxi, China

**Keywords:** 3T3-L1 adipocytes, insulin resistance, insulin signaling, krill oil, network pharmacology, untargeted lipidomics, untargeted metabolomics

## Abstract

Insulin resistance is a major contributor to metabolic disorders. We compared krill oil (KO), fish oil (FO), and soybean lecithin (SL) using untargeted metabolomics (UHPLC-HRMS), untargeted lipidomics (UPLC-QTOF-MS), network pharmacology, molecular docking, and a TNF-alpha-induced 3T3-L1 adipocyte model. Metabolomics detected 3,314 annotated features. In the KO versus FO comparison, 378 features were up-regulated and 593 were down-regulated in positive-ion mode, whereas 365 were up-regulated and 925 were down-regulated in negative-ion mode. Lipidomics detected 4,667 lipid-associated features and revealed distinct product-specific patterns of phospholipids, free fatty acids, glycerolipids, and sphingolipids. Network pharmacology identified 394 intersecting candidate targets, with AKT1, IL6, PPARG, and SRC prioritized as high-centrality nodes. Molecular docking was used to prioritize predicted component-target interactions. In mature 3T3-L1 adipocytes, TNF-alpha reduced insulin-stimulated 2-NBDG uptake. KO, FO, and SL increased glucose uptake relative to the TNF-alpha plus insulin group, with KO showing the largest response among the tested oils. KO also increased the *p*-AKT/AKT ratio and altered the p-IRS1/IRS1 ratio. These findings support compositional differences among the tested oils and cell-level modulation of insulin signaling by KO. They do not establish *in vivo* efficacy, direct target engagement, or an effect attributable solely to phospholipid form because EPA/DHA exposure was not dose-matched.

## 1 Introduction

Insulin resistance, as a core pathological feature of metabolic syndrome, type 2 diabetes mellitus, obesity, and related cardiovascular diseases (1, 2), is characterized by reduced responsiveness of peripheral tissues to insulin, thus leading to impaired glucose and lipid homeostasis (3, 4). Besides, the global prevalence of metabolic disorders continues to rise, posing a substantial burden on public health (5). Although lifestyle interventions and pharmacological treatments (such as metformin and thiazolidinediones) have been applied, their long-term adherence and side effects remain challenging (6, 7). Therefore, identifying natural dietary components with multi-target regulatory activities to improve insulin resistance and metabolic disorders has become a research hotspot.

Marine-derived omega-3 polyunsaturated fatty acids, particularly eicosapentaenoic acid (EPA) and docosahexaenoic acid (DHA), have been widely recognized for their beneficial effects on insulin sensitivity, lipid metabolism, and inflammation (8, 9). Fish oil (FO), as the most common source of EPA and DHA, is primarily in the form of triglycerides or ethyl esters. In contrast, krill oil (KO) derived from Euphausia superba contains EPA and DHA mainly incorporated into phospholipids and also contains the potent antioxidant astaxanthin (10). This unique phospholipid-bound omega-3 structure is considered to possess higher bioavailability and distinct biological activities compared with FO (11). Soybean lecithin (SL) is another widely used source of phospholipids, mainly composed of phosphatidylcholine but without omega-3 polyunsaturated fatty acids (12). Although comparative studies on the lipid profiles and metabolic effects of the krill oil and the fish oil have been reported, a comprehensive comparison of untargeted metabolites and lipidomes among the krill oil, fish oil, and soybean lecithin has not yet been reported. Furthermore, the precise molecular mechanisms by which the krill oil improves insulin resistance and metabolic disorders remain to be systematically elucidated (13). While previous comparative studies have primarily focused on bioavailability, fatty acid incorporation, or specific lipid-class differences between krill oil and fish oil, these investigations have largely relied on targeted approaches and have not systematically integrated global metabolomic and lipidomic profiling. Furthermore, head-to-head comparisons of KO with a plant-derived phospholipid source such as soybean lecithin – – which provides a phospholipid backbone without omega-3 polyunsaturated fatty acids – – have been notably absent. This study provides three key advances beyond previous work. First, it offers the first integrated, unbiased comparison of KO, FO, and SL using both untargeted metabolomics and untargeted lipidomics, enabling comprehensive molecular profiling without preconceived bias toward specific lipid classes. Second, it incorporates network pharmacology and molecular docking to predict potential multi-target mechanisms of KO, thereby bridging compositional differences with functional hypotheses. Third, it includes *in vitro* cellular validation in a TNF-α-induced insulin-resistant 3T3-L1 adipocyte model, which experimentally tests the predicted insulin-sensitizing activity and compares the cellular effects of the three oils under the same experimental conditions. This integrated workflow – – from compositional profiling to pathway prediction to cellular functional assessment – – distinguishes our study from previous comparative investigations and provides a more systematic molecular basis for understanding KO’s potential role in metabolic regulation (14).

In recent years, advances in untargeted metabolomics and lipidomics have enabled global analysis of small molecules and lipid species in complex biological matrices, providing an unbiased perspective for revealing compositional differences (15, 16). Simultaneously, network pharmacology which integrates target prediction, protein interaction networks, and pathway enrichment analysis, has emerged as a powerful tool for predicting the multi-target mechanisms of natural products (17, 18). Collectively, by combining multi-omics with network pharmacology, it is possible to link chemical composition to potential therapeutic effects.

Therefore, this study employed an integrated approach using untargeted metabolomics (UHPLC-HRMS), untargeted lipidomics (UPLC-QTOF-MS), and network pharmacology with the following aims: first, to systematically compare the metabolite and lipid profiles of the krill oil, fish oil, and soybean lecithin; second, to identify the key differential components characteristic of the krill oil; third, to predict the potential targets and signaling pathways involved in the intervention of the krill oil in insulin resistance and metabolic disorders. The resulting findings provide molecular-level evidence for the compositional uniqueness of the krill oil and offer a mechanistic basis for its functional properties superior to fish oil and soybean lecithin in metabolic regulation.

## 2 Results and discussion

### 2.1 Untargeted metabolomics analysis of KO, FO and SL

Untargeted metabolomics analysis was performed on three oil samples – krill oil (KO-A), deep-sea fish oil (FO-B), and soybean lecithin (SL-C) – to explore their differences. To comprehensively cover non-volatile metabolites with various polarities and ionization characteristics, detection was carried out in both positive and negative electrospray ionization modes (ESI^+^/ESI^–^). After database matching, a total of 3,314 metabolites were detected, including 1,503 in positive ion mode and 1,811 in negative ion mode.

To evaluate data stability and overall metabolic differences among samples, correlation analysis and principal component analysis (PCA) were performed on all samples. In both positive ion mode (Figure 1A) and negative ion mode (Figure 1B), samples within the same group showed extremely high correlations (r = 0.964–0.993), while correlations between different oil types were significantly lower (r = 0.075–0.301), indicating sound sample repeatability and marked differences in metabolic composition among the three oil systems. Notably, the correlation between deep-sea fish oil (B) and soybean lecithin (C) was the lowest, suggesting the largest difference in lipid composition between these two. This observation aligns with the known distinct chemical natures of triacylglycerol-rich fish oil and phospholipid-rich lecithin (19). PCA results further confirmed this trend (Figures 1C, D). In the positive ion mode, PC1 and PC2 explained 47.45% and 43.84% of the total variance, respectively; in the negative ion mode, they explained 49.37% and 43.98% of the total variance, respectively. The three oil samples were distinctly separated without overlap in the two-dimensional score plots, with krill oil (A), deep-sea fish oil (B), and soybean lecithin (C) each clustering in different regions, indicating significant differences in their overall lipid metabolic profiles. Meanwhile, samples within each group clustered tightly with no outliers observed, confirming the stable and reliable detection system and the high-quality data. The clear separation among the three oils suggests that the source of oil (marine animal vs. plant) and the chemical form of lipids (phospholipid-bound vs. neutral triglyceride) are the dominant factors driving the metabolic profile divergence (20).

**FIGURE 1:**
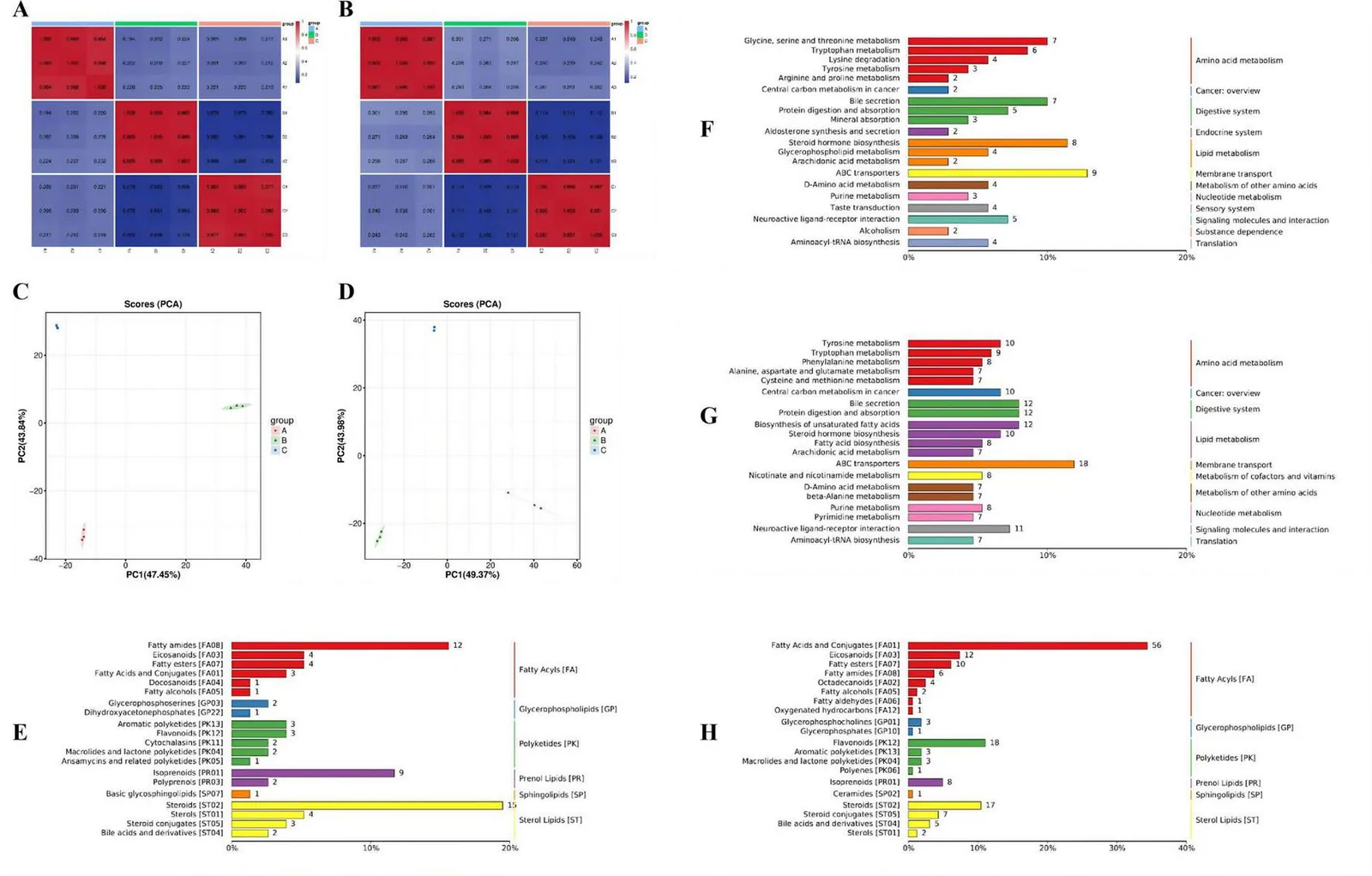
Analysis of the total sample. Correlation analysis in positive ion mode **(A)** and negative ion mode **(B)**; PCA analysis in positive ion mode **(C)** and negative ion mode **(D)**; KEGG pathway annotation in positive ion mode **(E)** and negative ion mode **(F)**; LIPID MAPS pathway annotation in positive ion mode **(G)** and negative ion mode **(H)**.

To elucidate the potential biological functions of the identified lipid metabolites in the samples, Kyoto Encyclopedia of Genes and Genomes (KEGG) pathway annotation and Lipid Metabolites and Pathways Strategy (LIPID MAPS) classification analysis were performed. In the KEGG pathway annotation, the metabolites, in the positive ion mode (Figure 1E), were mainly enriched in amino acid metabolism (glycine, serine and threonine metabolism; tryptophan metabolism; lysine degradation; arginine and proline metabolism), lipid metabolism (steroid hormone biosynthesis; glycerophospholipid metabolism; arachidonic acid metabolism), and membrane transport (ABC transporters), along with signaling regulation processes such as neuroactive ligand–receptor interaction and taste transduction. In the negative ion mode (Figure 1F), the enrichment trend was more pronounced. In addition to amino acid metabolism, membrane transport, and lipid metabolism, the proportions of digestive system and signaling molecules and interaction increased significantly, with ABC transporters showing the highest enrichment level, indicating that the sample lipidome was closely related to membrane structure composition and lipid transport. The prominence of ABC transporters in the negative ion mode enrichment analysis is particularly noteworthy, as these transporters are responsible for the cellular efflux of various lipids and xenobiotics, and their enrichment suggests that the oil samples, especially KO, may contain lipid species that are natural substrates or modulators of ABC transporters (9). Furthermore, the detection of arachidonic acid metabolism in both ionization modes implies that the oils differ not only in their fatty acid profiles but also in their potential to influence eicosanoid signaling pathways (17, 21).

The LIPID MAPS classification results further revealed the characteristics of the lipid composition. In the positive ion mode (Figure 1G), the metabolites were mainly distributed among fatty acyls, prenol lipids, and sterol lipids, with sterol lipids accounting for the highest proportion, suggesting an abundance of sterol-based lipids in the samples. In the negative ion mode (Figure 1H), fatty acyls constituted the most predominant lipid class, with fatty acids and conjugates being particularly dominant, followed by sterol lipids and polyketides, reflecting the abundance of long-chain polyunsaturated fatty acids and their derivatives in the samples. The complementary nature of the two ionization modes is evident: positive ESI preferentially detects sterol lipids and glycerophospholipids, while negative ESI is more sensitive to free fatty acids and their oxidized derivatives. This complementarity is well documented in lipidomics studies and underscores the importance of using both modes for comprehensive lipid coverage (15).

Combined KEGG and LIPID MAPS analysis indicated that the metabolites of the oil samples in this study were primarily involved in biological processes related to lipid metabolism, membrane structure construction, and signal regulation. The negative ion mode tended to resolve structural lipids such as fatty acids, while the positive ion mode was more favorable for detecting complex lipid molecules such as sterols and glycerophospholipids.

### 2.2 Differential metabolites and metabolite pathway enrichment of lipid systems

To systematically compare the metabolic differences between krill oil and deep-sea fish oil (A vs. B) as well as between krill oil and soybean lecithin (A vs. C), multivariate statistical analysis were performed in both positive and negative ion modes. Based on the screening criteria (VIP > 1, |log_2_FC| > 1, and *p* < 0.05), volcano plot analysis of differential metabolites between different oils were performed in both positive ion mode (ESI^+^) and negative ion mode (ESI^–^). It is important to note a methodological consideration regarding the differential metabolite screening in our multi-omics analysis. A stringent threshold of | log_2_FC| > 1 (in addition to VIP > 1 and *p* < 0.05) was applied across all comparisons. The rationale for this strict criterion was to prioritize the most robust, predominant, and structural compositional differences distinguishing the three distinct oil matrices, while effectively minimizing the false-positive noise inherent in high-throughput untargeted omics datasets. In the positive ion mode (ESI^+^), in A vs. B, 378 significantly up-regulated metabolites and 593 down-regulated metabolites were detected, while the remaining 532 metabolites showed no significant changes (Figure 2A). In A vs. C, 396 up-regulated metabolites and 501 down-regulated metabolites were screened, with 606 metabolites showing no significant changes (Figure 2C). In the negative ion mode (ESI^–^), further volcano plot analysis of differential metabolites among various oils were performed. In A vs. B, 365 significantly up-regulated metabolites and 925 down-regulated metabolites were detected, with 521 metabolites showing no significant changes (Figure 2B). In A vs. C, 469 up-regulated metabolites and 735 down-regulated metabolites were screened, with 607 metabolites showing no significant changes (Figure 2D). Overall, the number of down-regulated metabolites identified in the negative ion mode was significantly higher than that in the positive ion mode, indicating that the negative ion mode was more sensitive to changes in fatty acids and their derivatives, while the positive ion mode was more effective for detecting differences in polar lipids and certain signaling lipids. Both the two ionization modes showed sound complementarity, collectively indicating that the krill oil exhibited a systematic lipid remodeling feature compared to deep-sea fish oil and soybean lecithin. This difference did not result from the changes in a single metabolite but rather represented a structural alteration at the overall lipidome level. This allowed us to isolate core signature components for subsequent network pharmacology and molecular docking analyses. We acknowledge that this strict cutoff may exclude low- to moderate-abundance metabolites – particularly trace signaling lipids or metabolic intermediates – that could exert significant biological functions despite subtle concentration changes. Future targeted quantitative lipidomics studies are warranted to further characterize these potentially bioactive low-abundance species.

**FIGURE 2:**
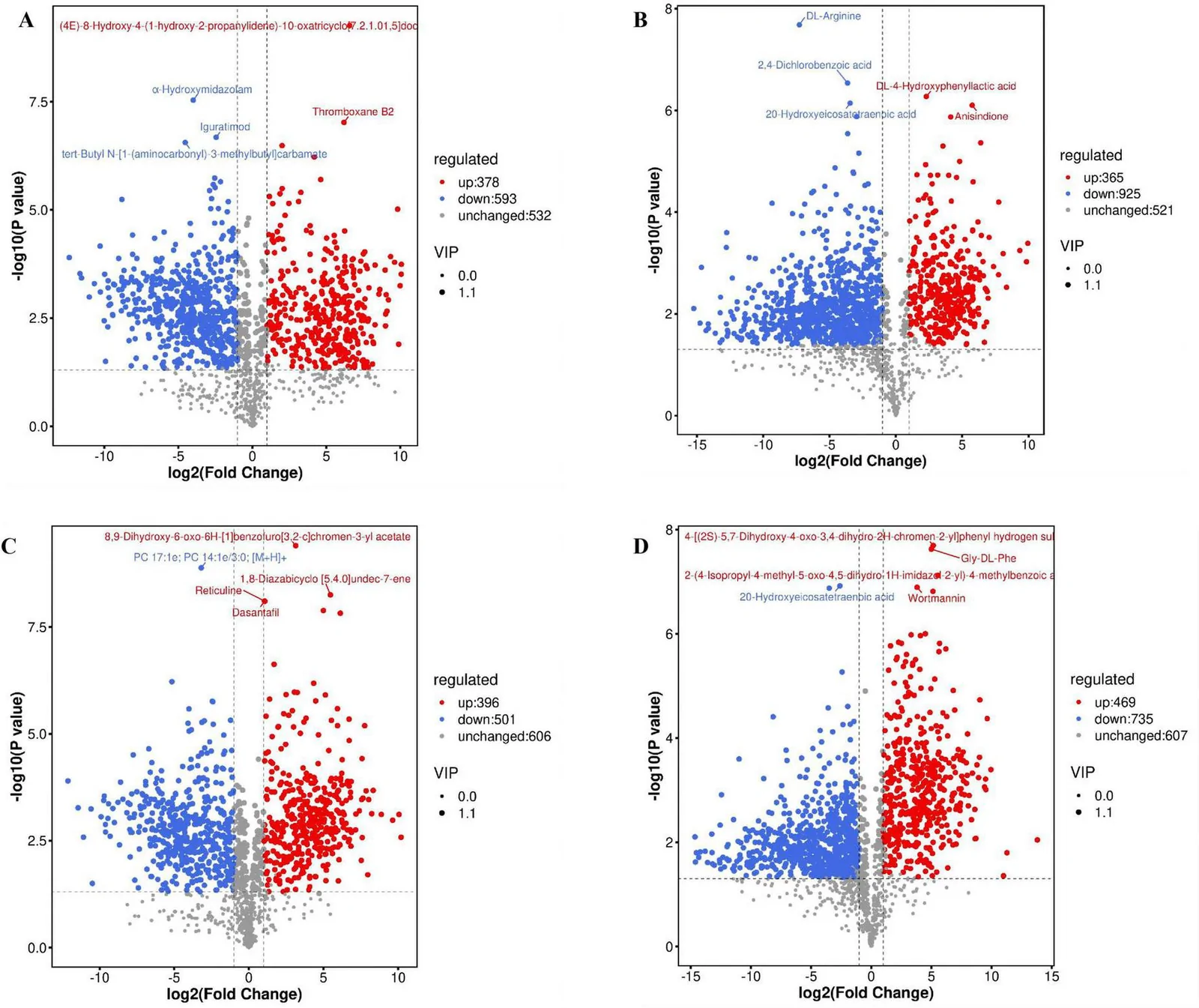
Analysis of differential metabolites. Volcano plot analysis was A vs. B in positive ion mode **(A)** and negative ion mode **(B)**; Volcano plot analysis was A vs. C in positive ion mode **(C)** and negative ion mode **(D)**.

KEGG network enrichment analysis further revealed the functional association characteristics of differential metabolites between krill oil (KO) and both deep-sea fish oil (FO) and soybean lecithin (SL). As to the KO vs. FO comparison, in the positive ion mode, differential metabolites were mainly enriched in purine metabolism (ko00230), tyrosine metabolism (ko00350), lysine biosynthesis (ko00300), arachidonic acid metabolism (ko00590), and ABC transporters (ko02010) (Supplementary Figure 1). Purine metabolism has been increasingly recognized as a key player in metabolic disorders: elevated serum uric acid, the end product of purine catabolism, is associated with insulin resistance, obesity and type 2 diabetes, although it remains debated whether uric acid is a causal factor or merely a marker of metabolic distress (22). Tyrosine metabolism also shows strong links to insulin resistance, as plasma tyrosine levels are elevated in insulin-resistant states and serve as predictive markers for incident type 2 diabetes (23). The enrichment of these two pathways in the KO vs. FO comparison suggests that krill oil and fish oil differentially modulate amino acid and nucleotide metabolism beyond their well-recognized effects on lipid homeostasis.

In the negative ion mode, differential metabolites were further concentrated in lipid signaling and steroid-related pathways, including arachidonic acid metabolism (ko00590), steroid hormone biosynthesis (ko00140), ovarian steroidogenesis (ko04913), primary bile acid biosynthesis (ko00120), and bile secretion (ko04976) (Supplementary Figure 1). Arachidonic acid metabolism is of particular interest, as arachidonic acid-derived eicosanoids are central mediators of inflammation. Pro-inflammatory eicosanoids (such as specific prostaglandins and epoxides) are associated with higher HOMA-IR, while anti-inflammatory resolvins derived from omega-3 fatty acids are linked to lower HOMA-IR (24). Chronic activation of eicosanoid pathways in metabolic syndrome drives persistent inflammation that exacerbates insulin resistance and dyslipidemia (20). Moreover, bile acids have emerged as key signaling molecules that regulate glucose and lipid metabolism through nuclear receptors such as FXR and TGR5, and dysregulated bile acid homeostasis is closely linked to insulin resistance and type 2 diabetes mellitus (23, 25). The enrichment of these bile acid-related pathways suggests that the two oils may differentially influence enterohepatic circulation and bile acid-mediated metabolic regulation. Therefore, in KO vs. FO, a lipid signaling network centered on arachidonic acid metabolism and extending to bile acid signaling regulates the network.

In the KO vs. SL comparison, in the positive ion mode, differential metabolites were mainly enriched in protein digestion and absorption (ko04974), aminoacyl-tRNA biosynthesis (ko00970), d-amino acid metabolism (ko00473), mineral absorption (ko04978), and tryptophan metabolism (ko00380) (Supplementary Figure 1). In the negative ion mode, a network centered on neurotransmitter-related metabolism was formed, including tryptophan metabolism (ko00380), serotonergic synapse (ko04726), arginine biosynthesis (ko00220), and central carbon metabolism in cancer (ko05230), and interconnected with arachidonic acid metabolism (ko00590) (Supplementary Figure 1). ABC transporters, which were enriched in both ionization modes in the KO vs. FO comparison, are transmembrane proteins that hydrolyze ATP to drive the transport of lipids, sterols, and xenobiotics across cellular membranes. They play critical roles in cholesterol and phospholipid efflux, reverse cholesterol transport, and the regulation of plasma membrane lipid composition (26). The enrichment of ABC transporters in the differential metabolite pathways suggests that the compositional differences between KO and FO may extend to their interactions with these key transport systems, potentially affecting cellular lipid homeostasis and signaling.

In summary, KO, FO, and SL exhibited systematic metabolic differentiation beyond fatty acid composition. FO and SL clustered together, whereas KO stood apart: FO was associated with inflammatory signaling, while KO favored energy metabolism and transmembrane transport. Compared to FO, KO uniquely regulated lipid signaling and energy intermediates; compared to SL, KO influenced amino acid and neurotransmitter metabolism. Collectively, the phospholipid-bound omega-3 fatty acids in KO enhance tissue delivery of EPA/DHA and actively reprogram metabolic networks toward improved energy homeostasis and reduced inflammation, providing a mechanistic basis for its superior benefits over FO and SL.

### 2.3 Untargeted lipids analysis of KO, FO and SL

Untargeted lipidomics analysis was further employed to investigate the differences among krill oil (KO), deep-sea fish oil (FO), and soybean lecithin (SL). Untargeted lipidomics is an emerging omics approach that enables the global, unbiased characterization of lipids in complex biological matrices, making it an ideal tool for compositional comparison among oils from different sources. The three oil samples were analyzed in both positive and negative ion modes (ESI^+^/ESI^–^), followed by database matching. A total of 4,667 metabolites were detected, including 2,016 in the positive ion mode and 2,651 in the negative ion mode.

PCA was used to analyze the overall lipid profiles. In the positive ion mode, PC1 and PC2 explained 46.20% and 37.05% of the variance, respectively. The three oil samples formed three distinctly separated clusters in the score plot, indicating significant differences in the lipid composition among the three oils. Furthermore, the replicates within each group clustered tightly, demonstrating sound stability and reproducibility of the analytical data (Figure 3A). In the negative ion mode, PC1 and PC2 explained 41.55% and 35.59% of the variance, respectively. The sample distribution trend was largely consistent with that observed in the positive ion mode, also showing clear separation among the three groups (Figure 3B). These PCA results confirm that the oil source is the dominant factor driving the lipidomic divergence among KO, FO, and SL, and that the intrinsic compositional differences are sufficiently robust to be captured at the overall lipidome level.

**FIGURE 3:**
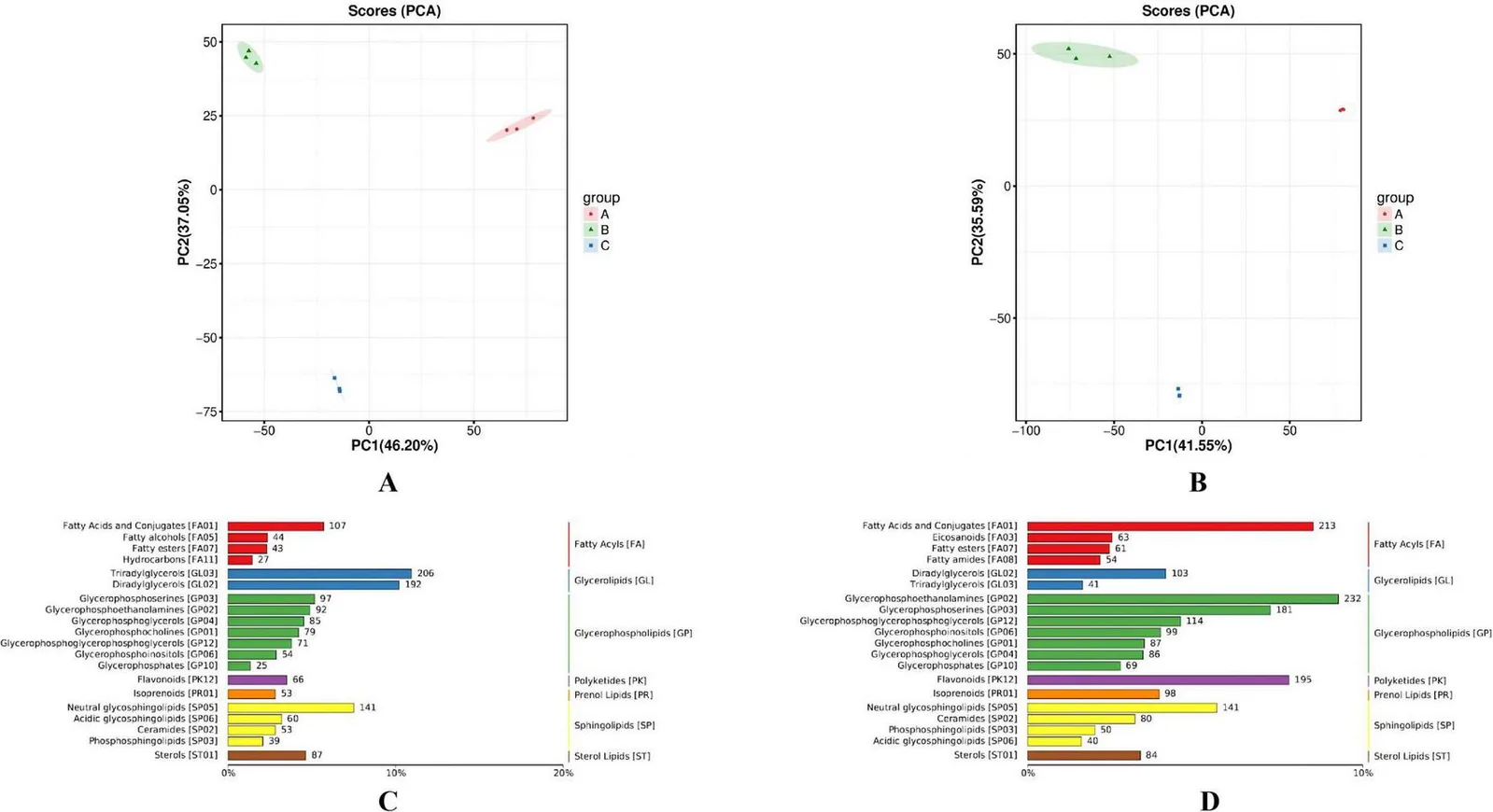
Comprehensive analysis of untargeted lipidomics. PCA analysis in positive ion mode **(A)** and negative ion mode **(B)**; LIPID MAPS pathway annotation in positive ion mode **(C)** and negative ion mode **(D)**.

LIPID MAPS (Lipid Metabolites and Pathways Strategy), as a classic lipid database and annotation platform, can systematically integrate lipid structure and metabolic pathway information and serve as an important resource for lipidomics research. LIPID MAPS provides a systematic and standardized approach to organizing lipid structural and biochemical data, and its nomenclature and classification system, has become the accepted community standard in the lipidomics field (27). In this study, the detected lipids were annotated based on the LIPID MAPS database, and the top 20 lipid categories with the highest annotation counts were selected for statistical and visualization analysis. As shown in Figure 3C, In the positive ion mode, the classification and annotation of lipid molecules based on the LIPID MAPS database revealed distinct compositional biases among different lipid categories. The top three most abundant lipid categories were glycerolipids (GL), glycerophospholipids (GP), and sphingolipids (SP), indicating that the sample lipidome was dominated by structural lipids and energy storage lipids. Notably, two specific lipid classes, triradylglycerols (GL03) and diradylglycerols (GL02), exhibited the highest metabolite counts, with 206 and 192, respectively, indicating the predominance of neutral energy-storage lipids. This was followed by neutral glycosphingolipids (SP05) (141), suggesting the important contribution of the lipids associated with membrane structure and signal transduction. As shown in Figure 3D, In the negative ion mode, the lipid class distribution exhibited a pronounced enrichment toward polar lipids. Glycerophospholipids (GP) were the most abundant lipid category, followed by fatty acyls (FA) and sphingolipids (SP). The enrichment of GP and SP classes in negative ion mode aligns with the known ionization preference of these lipid classes, as phosphate and carboxylic acid groups are more readily deprotonated under negative ESI conditions. Compared to the positive ion mode, the negative ion mode was more favorable for ionizing the lipids with strong polar functional groups such as phosphate, carboxylate, or sulfate groups, thus enabling a more comprehensive characterization of membrane structural lipids and signal transduction-related lipid molecules. The lipids were mainly concentrated in the categories such as glycerophosphoethanolamines (GP02), fatty acids and conjugates (FA01), glycerophosphoserines (GP03), neutral glycosphingolipids (SP05), and glycerophosphoglycerophosphoglycerols (GP12). The prominent detection of GP12 (cardiolipins and related lipids) in negative ion mode is particularly noteworthy, as cardiolipin is a signature lipid of the inner mitochondrial membrane and is difficult to ionize efficiently in positive mode. Its detectability here enhances the comparability of mitochondrial membrane lipid profiles across the three oil samples (28).

### 2.4 Core differential lipids analysis and their biological function network

Compared with group A (krill oil), the lipid profiles of group B (deep-sea fish oil) and group C (soybean lecithin) were more similar to each other, regardless of the ionization mode. Based on the screening criteria (VIP > 1, | log_2_FC| > 1, and *p* < 0.05), volcano plot analysis of differential lipid metabolites among different oils were performed in both positive ion mode (ESI^+^) and negative ion mode (ESI^–^).

#### 2.4.1 Volcano plot analysis of differential lipids

KO vs. FO (A vs. B): In the positive ion mode (Figure 4A), a total of 428 up-regulated and 932 down-regulated lipids were detected in KO relative to FO. The up-regulated metabolites in FO mainly included long-chain hydrocarbons and oxidized lipids (13-Methyl-1-tritriacontene, 3-hydroxy-decanoic acid), fatty acid derivatives and ethanolamides, bile acid-like structures, and phytooxylipins, suggesting enhanced lipid oxidation and degradation in FO. In contrast, KO was enriched in phosphatidylcholines (PC), phosphatidylglycerols (PG), phosphatidylserines (PS), phosphatidylinositols (PI), and cardiolipins (CL). In the negative ion mode (Figure 4B), KO vs. FO yielded 131 up-regulated and 1,135 down-regulated lipids. The top 10 differential lipids in FO were biased toward free fatty acids and their oxidized/epoxidized derivatives as well as eicosanoid-related signals (e.g., PGE_3_, 15-deoxy-Δ^12^,^14^-PGJ_2_), whereas those in KO were mainly concentrated in structural membrane lipids such as PG, DG, PC, and anionic phospholipids PI and PS. The presence of eicosanoid-related signals such as PGE_3_ and 15-deoxy-Δ^12^,^14^-PGJ_2_ in FO suggests that the free fatty acids in fish oil are more prone to enzymatic or non-enzymatic oxidation, potentially generating pro-inflammatory or pro-resolving mediators. In contrast, the enrichment of anionic phospholipids (PI, PS) and cardiolipin in KO indicates a higher proportion of membrane-structuring lipids. Cardiolipin, which exists almost exclusively in the inner mitochondrial membrane, plays critical roles in mitochondrial bioenergetics, including maintaining the structural integrity of respiratory chain complexes and facilitating electron transport (8, 29, 30). Furthermore, the presence of PS and PI in KO is noteworthy, as these anionic phospholipids are not merely structural components but also participate in signaling cascades. PS on the inner leaflet of the plasma membrane serves as a docking site for signaling proteins containing pleckstrin homology domains, and its externalization acts as an “eat-me” signal in apoptosis. PI, particularly its phosphorylated derivatives, is a central hub for membrane signaling, regulating processes ranging from cell growth to glucose transport. The enrichment of these bioactive phospholipids in KO suggests that krill oil may have inherent signaling-modulating potential beyond simple nutrient provision. The retention of PS and PI in a phospholipid-bound form may also confer higher stability and bioavailability compared to free fatty acid counterparts. Collectively, KO featured “enrichment of phospholipid-type ω-3 and anionic phospholipid backbones”, while FO featured “free fatty acids and eicosanoid/oxidized derivative lipid signaling”.

**FIGURE 4:**
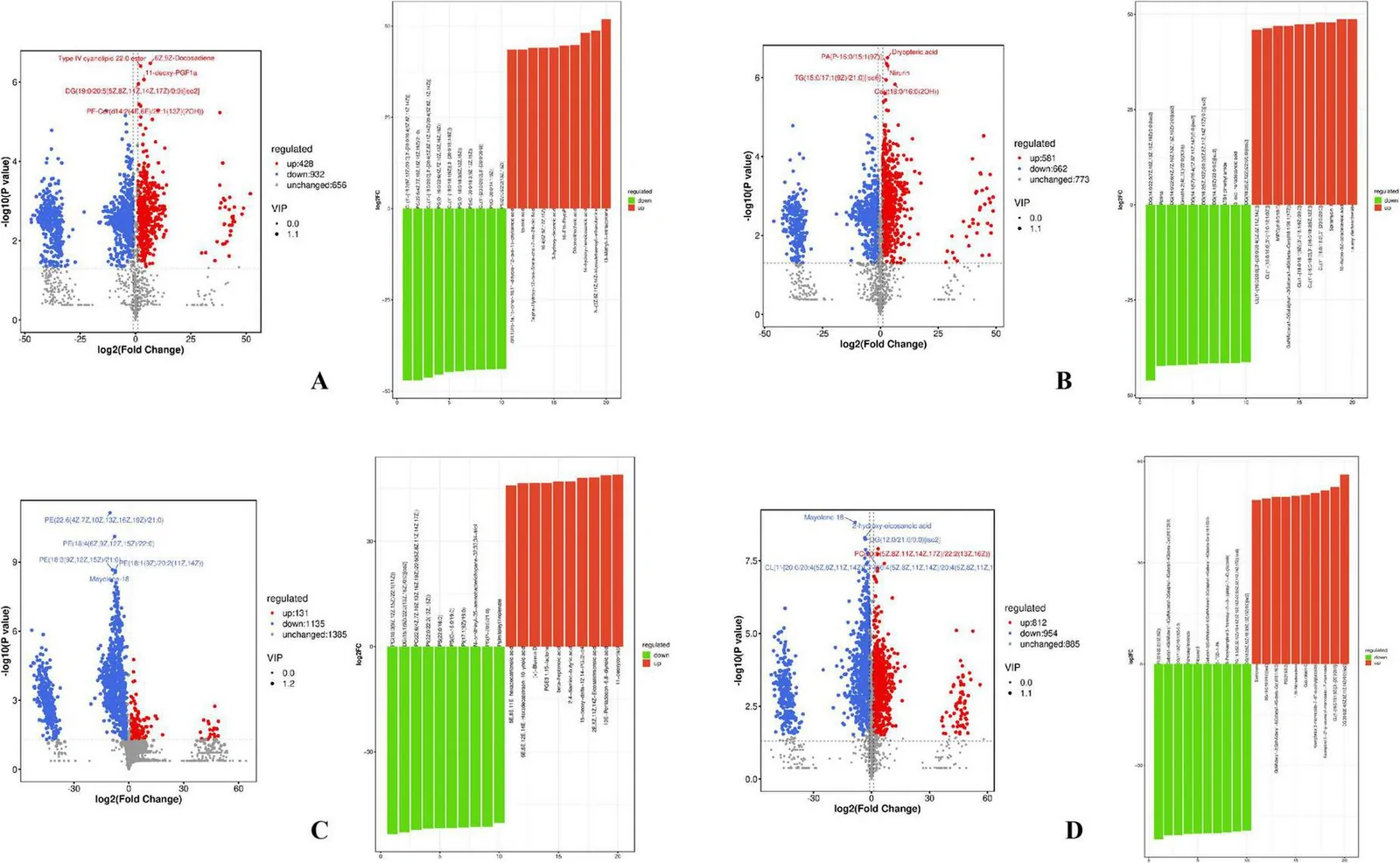
Integrated volcano plot and fold change (FC) bar plot analyses of differential lipids in krill oil (KO) vs. fish oil (FO) and KO vs. soybean lecithin (SL). **(A)** KO vs. FO in positive ion mode; **(B)** KO vs. FO in negative ion mode; **(C)** KO vs. SL in positive ion mode; **(D)** KO vs. SL in negative ion mode.

KO vs. SL (A vs. C): In the positive ion mode (Figure 4C), a total of 1,243 significantly different lipid metabolites were identified, including 581 up-regulated and 662 down-regulated in KO relative to SL. The top 10 enriched lipids in KO were mainly energy storage or fatty acid metabolism intermediates such as diacylglycerols (DG), retinal, 3-oxo-nonadecanoic acid, and LTB4 dimethyl amide, which were nearly absent in SL. In the negative ion mode (Figure 4D), KO vs. SL showed 812 up-regulated and 954 down-regulated lipids. The up-regulated lipids in KO included diacylglycerols (DG), cardiolipins (CL), phosphatidylglycerols (PG), and glycosphingolipids containing GlcNAc structures. DG is a key intermediate in glycerophospholipid synthesis and remodeling (Kennedy pathway and Lands cycle), and its enrichment suggests enhanced membrane lipid renewal. DG is unique in lipid metabolism because it serves not only as an intermediate product for triglyceride synthesis, but also as a signaling molecule that activates proteins containing DG-responsive elements, such as protein kinase C (PKC). Consequently, DG acts as a hub between energy metabolism and intracellular signaling (9). Accumulation of certain DG species, particularly those containing arachidonic acid, has been implicated in the activation of novel PKC isoforms that impair insulin signaling. However, the DG species identified in our KO samples may represent distinct molecular species with different signaling outcomes. The enrichment of DG in KO points to a more dynamic lipid turnover system, potentially enabling rapid adaptation to metabolic demands. Furthermore, DG is a precursor for the synthesis of PI and PS via CDP-DAG pathways, so its abundance may indicate enhanced capacity for generating signaling-competent phospholipids (31). CL, a signature lipid of the inner mitochondrial membrane, indicates a bias toward energy metabolism-related membrane structures. CL deficiency or remodeling defects have been linked to mitochondrial dysfunction and metabolic disorders, including insulin resistance and type 2 diabetes (32). The enrichment of CL in KO suggests that krill oil may provide a lipid environment more conducive to optimal mitochondrial function, which is particularly relevant for improving energy homeostasis in insulin-resistant states. Elevated glycosphingolipids may promote lipid raft formation, affecting membrane signal transduction and inflammatory regulation. In contrast, SL showed higher contents of PI, PS, and some triglyceride/diacylglycerol derivatives, which are mostly related to membrane structural stability and phospholipid storage. Thus, krill oil exhibits a “functional membrane lipid network” rich in ω-3 phospholipids and sphingolipids, whereas soybean lecithin appears as a “structural storage-type lipid system” dominated by PC. This structural difference may underlie their distinct bioavailability, membrane integration efficiency, and physiological functions.

#### 2.4.2 KEGG pathway enrichment analysis

KO vs. FO: In the positive ion mode (Figure 5A), differential lipids between KO and FO were mainly enriched in pathways related to lipid synthesis and sterol metabolism, including fatty acid biosynthesis (ko00061), fatty acid elongation (ko00062), fatty acid degradation (ko00071), steroid biosynthesis (ko00100), and beta-alanine metabolism (ko00410). (R)-3-Hydroxybutanoyl-CoA was higher in KO, suggesting more active fatty acid β-oxidation. Conversely, 4alpha-methylzymosterol (a sterol biosynthesis intermediate) was significantly higher in FO, indicating a higher level of steroid structural lipids in FO. The distinct metabolic signatures observed here indicate that KO and FO differentially engage the fatty acid metabolic network: KO is more “active” in the sense that it simultaneously upregulates synthesis and oxidation pathways, reflecting a high-turnover, flexible lipid system, whereas FO appears to be more associated with sterol-related lipid pools. This divergence may partly explain the distinct metabolic effects of the two oils *in vivo*. In the negative ion mode (Figure 5B), KO vs. FO differential lipids were enriched in butanoate metabolism (ko00650), fatty acid biosynthesis (ko00061), retinol metabolism (ko00830), steroid hormone biosynthesis (ko00140), and ubiquinone and other terpenoid-quinone biosynthesis (ko00130). Levels of lauric acid and myristic acid were significantly higher in KO than in FO, indicating that krill oil is richer in medium-chain saturated fatty acids (10, 12). Medium-chain fatty acids such as lauric acid and myristic acid are known to be more rapidly absorbed and preferentially oxidized rather than stored, making them metabolically distinct from long-chain fatty acids. Their enrichment in KO may contribute to the improved energy metabolism profile of krill oil. 2-Hydroxyglutaric acid was elevated in KO and participated in butanoate metabolism, suggesting more active energy metabolism and short-chain fatty acid metabolism. 2-Hydroxyglutaric acid is a metabolite that has gained significant attention due to its roles in epigenetics and metabolism. While traditionally associated with certain cancers as an “oncometabolite”, emerging evidence suggests that 2-HG also serves as a signaling molecule linking cellular metabolism to chromatin regulation. The elevation of 2-HG in KO is intriguing, as it may reflect altered flux through the TCA cycle or enhanced ketone body metabolism (33). This finding warrants further investigation into whether krill oil-derived lipid components can influence cellular epigenetic states via modulation of 2-HG levels. Additionally, vitamin K_2_ was significantly enriched in KO.

**FIGURE 5:**
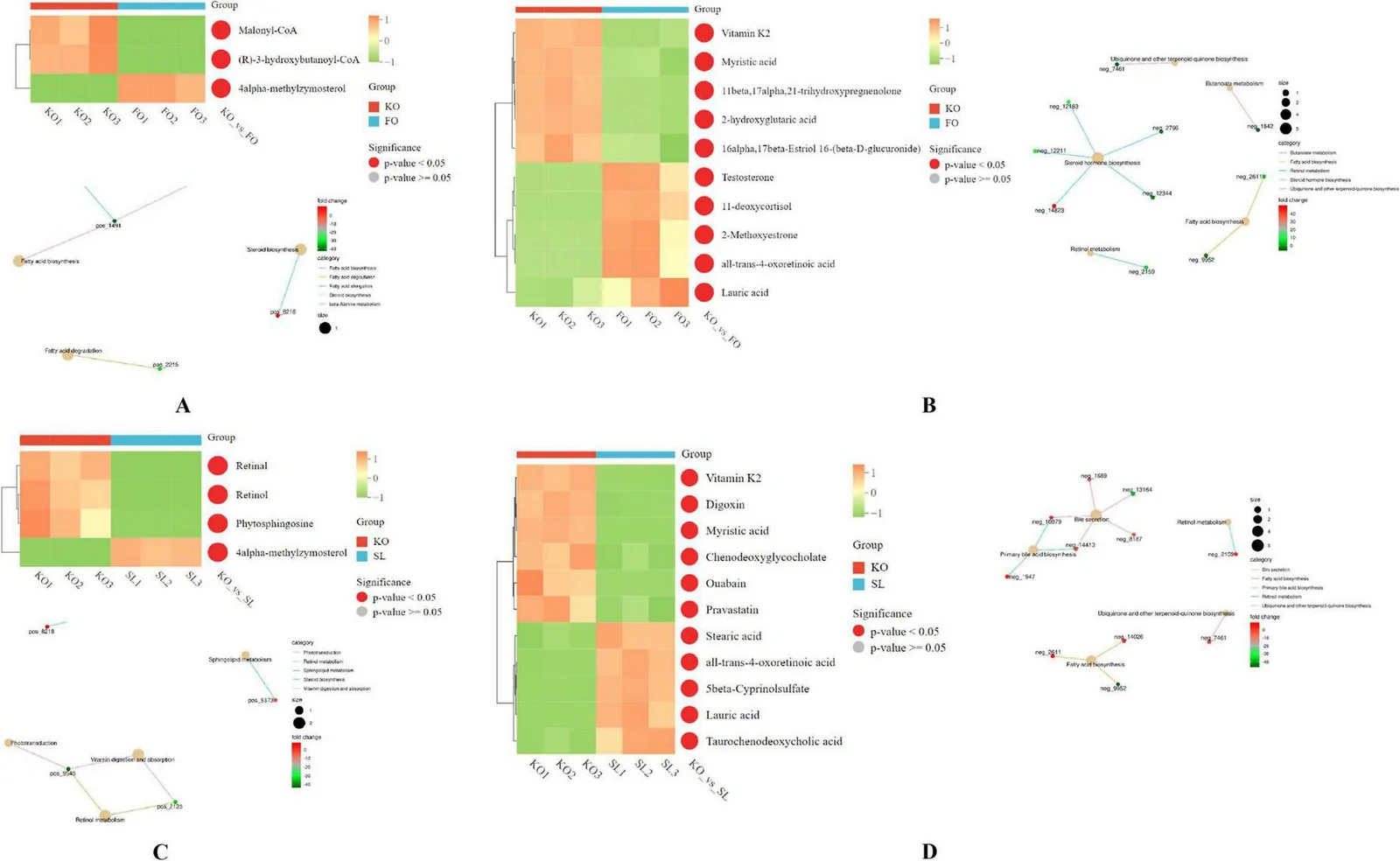
Heatmap and KEGG pathway enrichment network analysis of differential lipid annotations. **(A, B)** KO versus FO in positive- and negative-ion modes, respectively. **(C, D)** KO versus SL in positive- and negative-ion modes, respectively. In the heatmaps, rows represent differential lipid annotations and columns represent individual samples. The color scale indicates normalized relative abundance, with red and blue representing relatively higher and lower abundance, respectively. In the KEGG enrichment networks, nodes represent enriched pathways or associated lipid annotations, and edges represent pathway-feature associations. Node size indicates the number of mapped lipid annotations, whereas node color represents the enrichment significance according to the reported *P* value or FDR-adjusted *P* value.

KO vs. SL: In the positive ion mode (Figure 5C), retinal, retinol, and phytosphingosine were significantly higher in KO than in SL, being involved in retinol metabolism (ko00830) and sphingolipid metabolism (ko00600). Retinol and retinal are key active forms of vitamin A, essential for the visual cycle and epithelial differentiation; their enrichment in KO suggests that the krill oil system is more favorable for dissolving and stabilizing fat-soluble vitamins. Vitamin A derivatives are critical regulators of gene expression via nuclear retinoic acid receptors (RARs) and retinoid X receptors (RXRs). These receptors form heterodimers with PPARs and other metabolic nuclear receptors, thereby directly influencing glucose and lipid metabolism. The presence of retinol and retinal in KO, but not in SL, indicates that krill oil may provide not only omega-3 fatty acids but also essential cofactors for nuclear receptor-mediated metabolic regulation. Phytosphingosine, a sphingolipid backbone precursor, participates in membrane lipid raft construction and signal transduction; its high abundance in KO implies that krill oil may more readily form structured lipid delivery systems. Phytosphingosine is not merely a structural lipid but also a bioactive molecule with documented anti-inflammatory and anti-cancer properties. Its abundance in KO suggests that krill oil contains diverse signaling-competent lipids beyond the well-characterized EPA and DHA. This multi-component signaling potential may contribute to the superior metabolic benefits of krill oil. Compared with SL, KO not only alters fatty acid composition but also significantly enhances the bioavailability potential of fat-soluble vitamins and membrane structural lipids, which may explain the higher physiological activity of phospholipid-type ω-3 lipids. In the negative ion mode (Figure 5D), KO vs. SL differential lipids were mainly enriched in bile secretion (ko04976), fatty acid biosynthesis (ko00061), primary bile acid biosynthesis (ko00120), retinol metabolism (ko00830), and ubiquinone and other terpenoid-quinone biosynthesis (ko00130). Bile acids have emerged as key signaling molecules that regulate glucose and lipid metabolism through the nuclear receptor FXR and the G-protein-coupled receptor TGR5. Dysregulated bile acid homeostasis is closely linked to insulin resistance and type 2 diabetes. The enrichment of bile secretion and primary bile acid biosynthesis pathways in KO vs. SL suggests that KO has a higher capacity to interact with enterohepatic circulation and bile acid-mediated signaling. Compared with SL, KO had significantly higher levels of fat-soluble functional factors, especially vitamin K_2_ and retinol/retinal precursors, indicating a stronger capacity of KO as a lipid matrix to carry fat-soluble active ingredients. Vitamin K_2_ has garnered increasing attention for its extra-skeletal roles, including modulation of mitochondrial function, inflammation, and insulin sensitivity. The presence of vitamin K_2_ in KO, uniquely among the three oils examined, suggests that krill oil is a rare marine source of this important bioactive lipid. In terms of fatty acid metabolism, KO retained structural fatty acid intermediates such as lauric acid and myristic acid, whereas these fatty acids were significantly elevated in SL, suggesting that the phospholipid system in SL is more prone to fatty acid turnover and remodeling, while the lipid composition in KO is more stable.

#### 2.4.3 Data integration and management of overlapping annotations

The metabolomics and lipidomics datasets were analyzed and interpreted as two complementary but platform-specific datasets. The UHPLC-HRMS metabolomics workflow provided broad coverage of polar and semi-polar small molecules and metabolic intermediates, whereas the UPLC-QTOF-MS lipidomics workflow provided more detailed characterization of lipid molecular species and lipid classes.

The detected-feature numbers from the two platforms were reported separately and were not summed. Overlapping class-level annotations, including PC, PG, and PI, were not counted as additional independent biological discoveries. Because the same lipid class may contain multiple molecular species and may be annotated differently according to ionization mode, adduct type, database coverage, and MS/MS evidence, class-level overlap was interpreted as platform-level concordance rather than direct duplication. Species-level lipid comparisons were based primarily on the lipidomics dataset. Integration was performed at the level of biological interpretation. Metabolomics data were used to evaluate broad metabolic patterns and pathway-associated small-molecule changes, while lipidomics data were used to characterize membrane-lipid composition, storage lipids, sphingolipids, cardiolipin-related species, and lipid remodeling. Concordant pathways or biological themes identified by both platforms were considered supportive cross-platform evidence, whereas platform-specific findings were interpreted according to the analytical strengths of each method.

### 2.5 Network pharmacology analysis of the KO’s intervention in insulin resistance and metabolic disorders

To explore the potential relationships between KO-associated components and insulin resistance, the 9 core differential components (selected based on statistical enrichment and lipid-class representativeness) and one unique component (astaxanthin) identified by the multi-omics analyses were subjected to network-pharmacology analysis. Disease-related targets associated with insulin resistance and metabolic disorders were collected from the GeneCards, OMIM, and TTD databases. SwissTargetPrediction and the Similarity Ensemble Approach database identified 441 predicted protein targets. After intersection with the disease-related targets, 394 overlapping candidate targets were obtained (Figure 6A).

**FIGURE 6:**
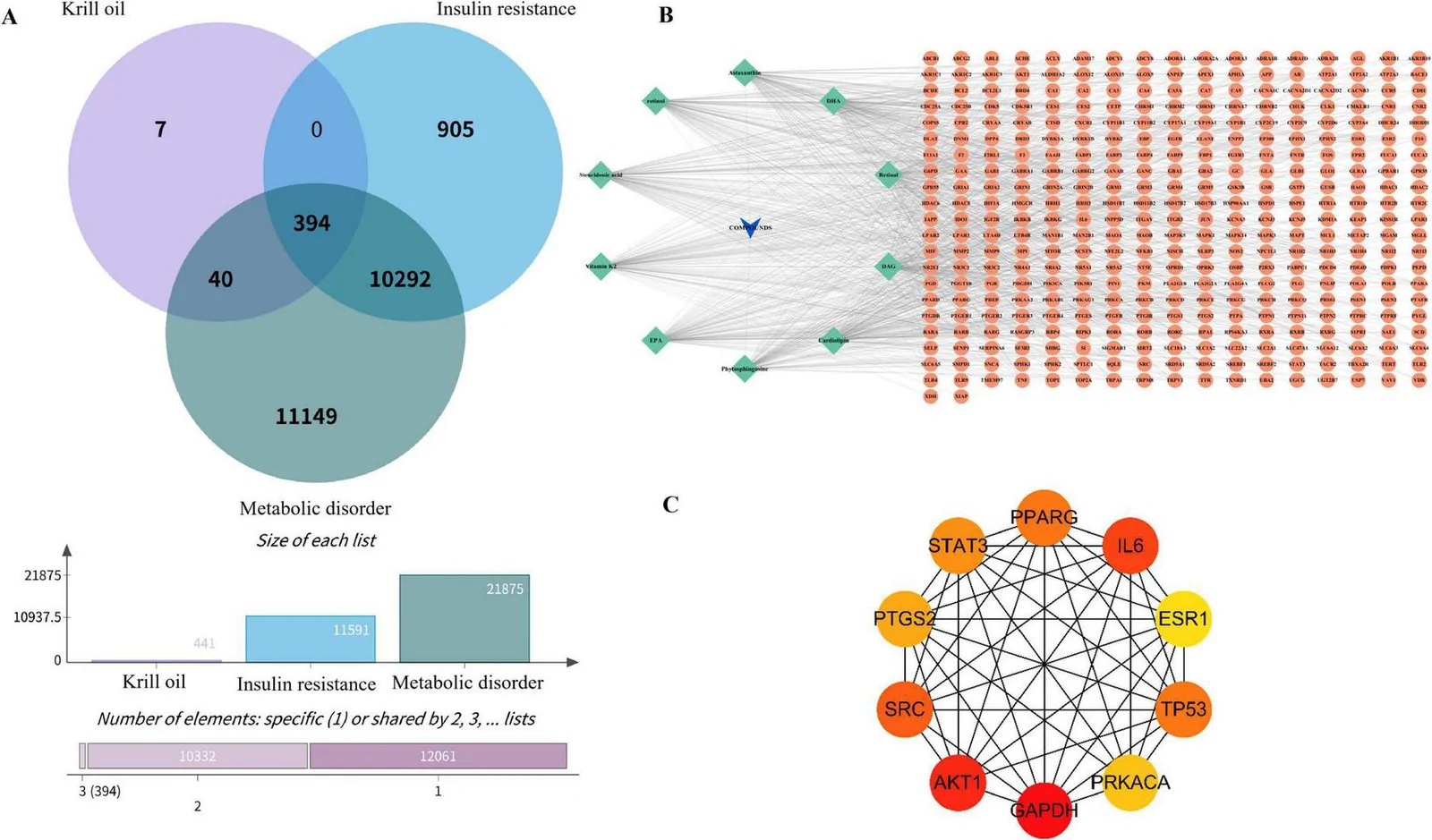
Network-pharmacology analysis of KO-associated component annotations related to insulin resistance and metabolic disorders. **(A)** Venn diagram showing the intersection between KO-associated component targets and disease-related targets. **(B)** Component-target-disease network constructed from nine core differential component annotations and one KO-specific component annotation. **(C)** Protein-protein interaction network of the overlapping candidate targets. Nodes represent components, targets, or disease-related terms, and edges represent predicted or database-derived associations.

The component-target-disease network constructed using Cytoscape 3.8.2 contained 407 nodes and 1,064 edges (Figure 6B). Topological analysis identified stearidonic acid, EPA, DHA, and DAG as highly connected component annotations (Table 1). These results indicate computational network prominence but do not establish pharmacological activity or direct component-target interactions. The 394 overlapping targets were submitted to the STRING database, generating a PPI network containing 390 nodes and 4,702 edges. The top-ranked nodes included GAPDH, AKT1, IL6, SRC, TP53, PPARG, STAT3, PTGS2, PRKACA, and ESR1 (Figure 6C). AKT1 and PPARG are associated with insulin signaling and adipocyte metabolism, whereas IL6, STAT3, and SRC are involved in inflammatory and metabolic regulation (34, 35). GAPDH showed high network centrality, but its ubiquitous expression, housekeeping function, and extensive database connectivity may contribute to this ranking. GAPDH was therefore not interpreted as a KO-specific validated target. The newly added cellular experiments showed that KO increased insulin-stimulated 2-NBDG uptake and the p-AKT/AKT ratio in TNF-alpha-treated 3T3-L1 adipocytes (36). These findings provide pathway-level functional evidence consistent with the involvement of AKT-related insulin signaling, but they do not establish AKT1 as a direct molecular target or demonstrate physical binding between AKT1 and individual KO components.

**TABLE 1:** Top 9 KO-associated component annotations in the component-target-disease network.

Molecule name	CID	Degree	Betweenness centrality	Closeness centrality
Stearidonic acid	5312508	123	0.039029	0.422477
EPA	446284	113	0.03016	0.413863
DHA	445580	105	0.028116	0.407222
DAG	190861	62	0.011923	0.374885
Cardiolipin	1.66E+08	60	0.011333	0.373505
Phytosphingosine	122121	30	0.002881	0.353967
Astaxanthin	5281224	30	0.002437	0.353967
Retinol	445354	24	0.000948	0.350302
Retinal	638015	22	0.000846	0.349097

GO enrichment analysis indicated that the candidate targets were highly associated with mitochondrial energy metabolism, including mitochondrial electron transport, ATP metabolism, purine nucleoside triphosphate metabolism, and NADH dehydrogenase activity (Figure 7A). Notably, these computational pathway predictions exhibit a striking and deep correlation with our empirical lipidomics findings. As observed in the lipidomic profiling, KO is uniquely enriched in cardiolipins (CL) compared to both FO and SL. Cardiolipin is an exclusive and essential structural phospholipid of the inner mitochondrial membrane, playing an irreplaceable role in stabilizing respiratory chain complexes (such as NADH dehydrogenase/Complex I) and facilitating efficient electron transport. Since mitochondrial dysfunction and impaired ATP synthesis are recognized hallmarks of insulin resistance, this cross-omics convergence offers a cohesive mechanistic insight. It suggests that KO may ameliorate metabolic disorders not only by modulating conventional kinase signaling but also by directly providing the critical structural lipids (like CL) necessary to restore mitochondrial membrane integrity and bioenergetics. Thus, the lipidomic composition of KO directly and physically supports the functional mitochondrial networks predicted by the GO enrichment (37, 38). KEGG enrichment analysis identified 146 pathways at *P* < 0.05, including insulin resistance, PPAR signaling, AMPK signaling, arachidonic acid metabolism, and regulation of lipolysis in adipocytes (Figure 7B). The recurrence of arachidonic acid metabolism in both the network-pharmacology and multi-omics analyses indicates concordance at the pathway-association level (39–41). However, enrichment analysis does not establish pathway activation, inhibition, or causal regulation by KO.

**FIGURE 7:**
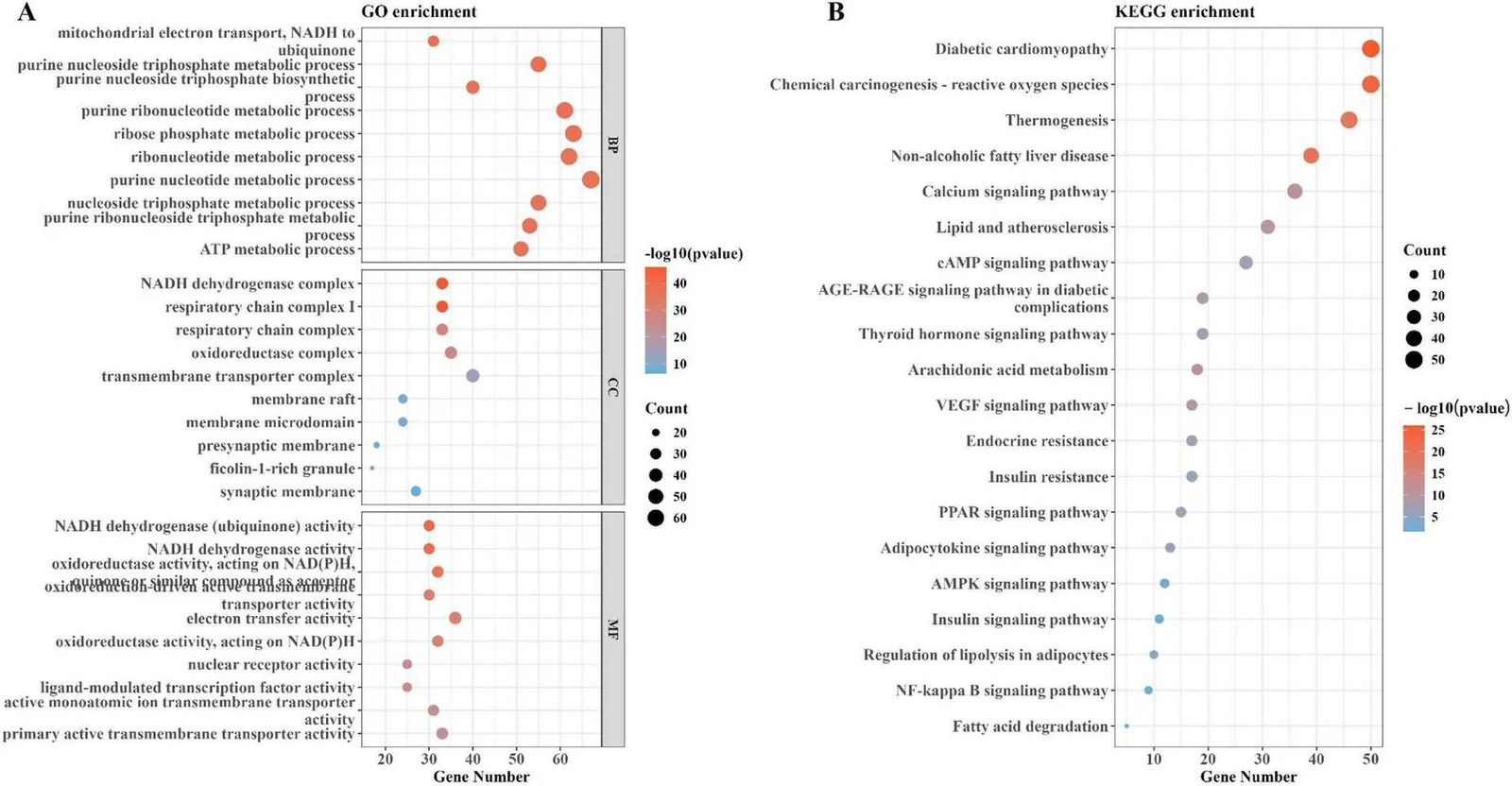
GO and KEGG enrichment analyses of candidate KO targets related to insulin resistance and metabolic disorders. **(A)** GO enrichment analysis involving biological process, cellular component, and molecular function categories. **(B)** KEGG pathway enrichment analysis. Dot size represents the number of mapped targets, and dot color represents the reported P value or FDR-adjusted *P* value.

### 2.6 The binding pattern between key active components and core targets and the molecular docking verification

Molecular docking was used to compare the predicted binding poses and docking scores of selected KO-associated component annotations against prioritized candidate targets. More negative docking scores indicated more favorable predicted ligand-receptor complementarity within the same computational protocol. The scores were used for candidate prioritization and hypothesis generation rather than as experimentally measured binding affinities (42, 43). The docking scores ranged from −3.74 to −7.73 kcal/mol (Figure 8A). Among the retained component-target pairs, DAG-AKT1 showed the most negative docking score (−7.73 kcal/mol), followed by DHA-AKT1 (−7.61 kcal/mol). EPA and stearidonic acid also showed predicted interactions with AKT1. Visualization of the predicted docking poses suggested potential hydrogen-bonding and hydrophobic contacts between the selected components and the modeled target pockets (Figures 8B–E). These contacts represent docking-model geometries rather than experimentally observed molecular interactions (44). Overall, the docking analysis identified candidate component-target relationships for further investigation. The docking scores and cellular phosphorylation results do not establish direct binding, target selectivity, or specific engagement of AKT1 by individual KO components. Direct target-engagement assays, such as SPR, CETSA, or another appropriate experimental approach, are required to verify these predicted interactions. Furthermore, we acknowledge the limitation that a positive control (e.g., an established AKT1 agonist or native ligand) was not included in the current docking simulation, which limits our ability to quantitatively benchmark the predicted binding affinities of the selected KO components.

**FIGURE 8:**
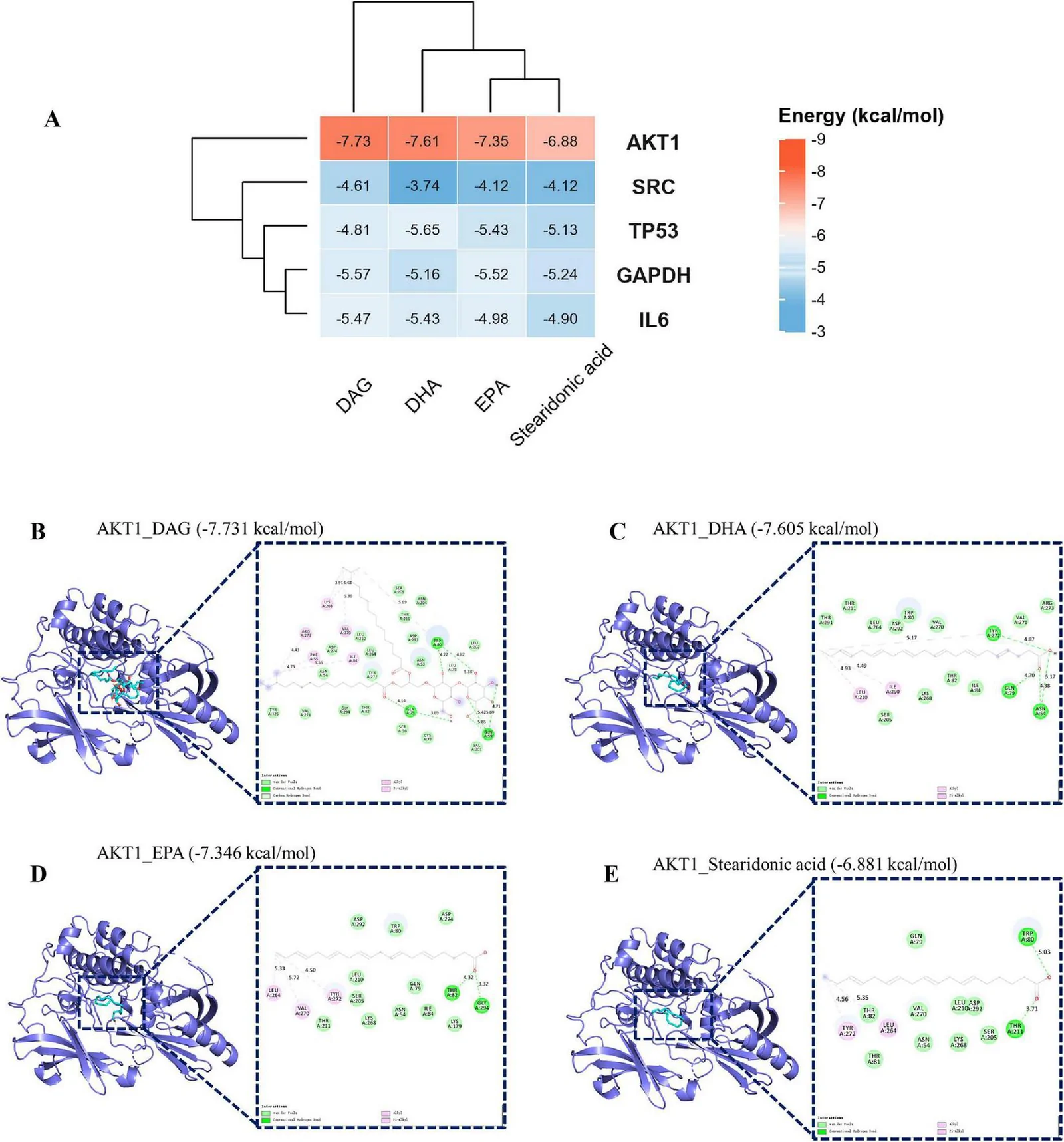
Predicted molecular docking poses and scores of selected component-target pairs. **(A)** Docking-score heatmap; **(B)** predicted docking pose of DAG with AKT1; **(C)** predicted docking pose of DHA with AKT1; **(D)** predicted docking pose of EPA with AKT1; and **(E)** predicted docking pose of stearidonic acid with AKT1.

### 2.7 *In vitro* validation of KO effects on insulin-resistant adipocytes

To experimentally assess the predicted anti-insulin-resistance potential of KO and compare its cellular effects with those of FO and SL. As shown in Figure 9A, insulin treatment significantly increased 2-NBDG uptake compared with the untreated control group (*P* < 0.01), confirming the insulin responsiveness of the differentiated adipocytes. TNF-alpha pretreatment markedly reduced insulin-stimulated glucose uptake compared with the insulin-only group (*P* < 0.01), consistent with the establishment of a cellular insulin-resistance model. Compared with the TNF-alpha plus insulin group, co-treatment with KO, FO, or SL significantly increased 2-NBDG uptake (*P* < 0.05 for all three treatments). Among the three tested oils, KO produced the largest increase, with glucose uptake significantly higher than that observed in the FO and SL groups (*P* < 0.05). These results indicate that KO alleviated TNF-alpha-associated impairment of insulin-stimulated glucose uptake under the present experimental conditions.

**FIGURE 9:**
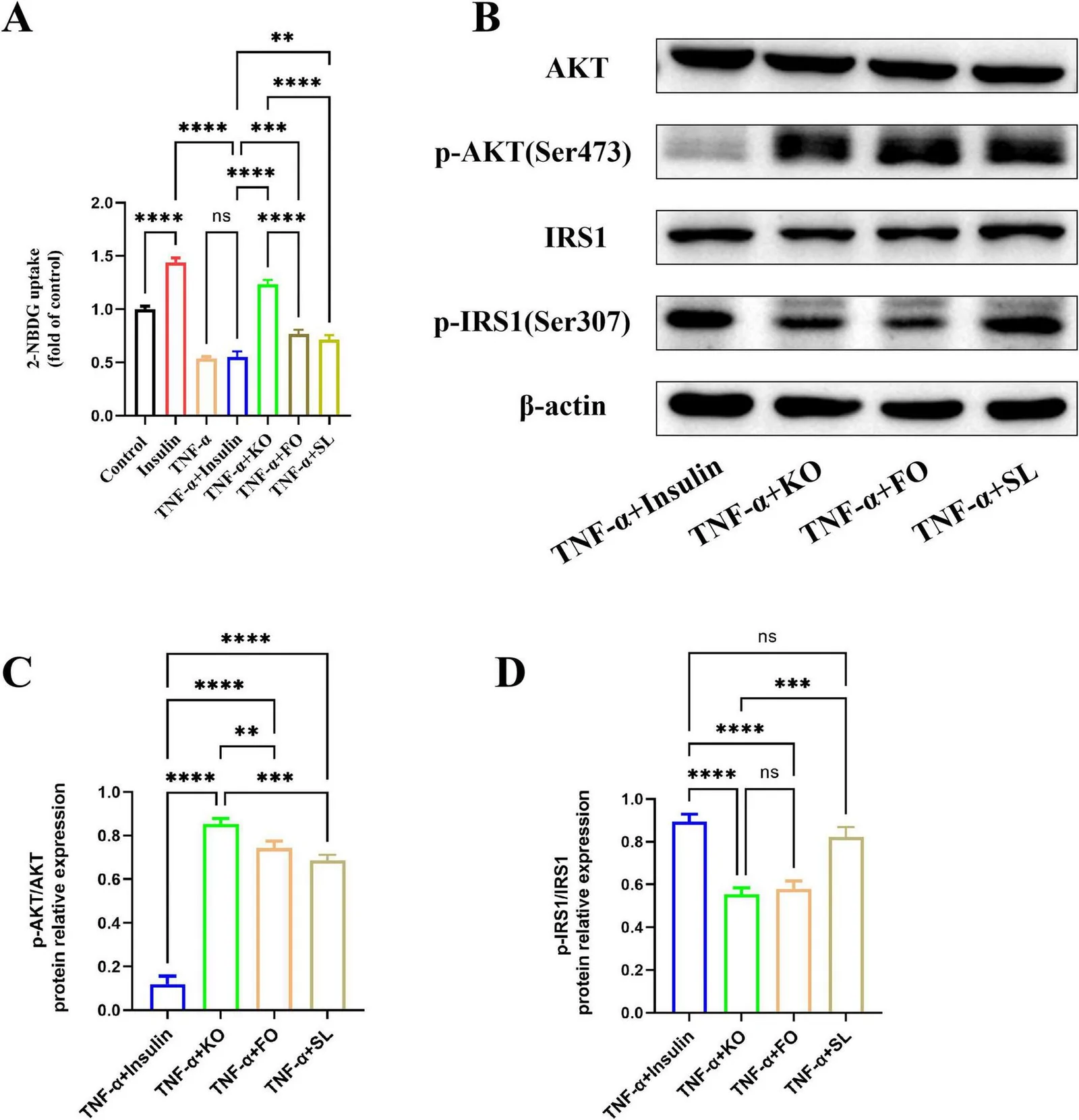
Effects of KO, FO, and SL on glucose uptake and insulin-signaling proteins in TNF-alpha-treated 3T3-L1 adipocytes. **(A)** 2-NBDG uptake in the indicated treatment groups. **(B)** Representative Western blot bands for total and phosphorylated AKT and IRS1. **(C)** Densitometric quantification of the p-AKT/AKT ratio. **(D)** Densitometric quantification of the p-IRS1/IRS1 ratio. ns indicates not significant (*P* ≥ 0.05); *P < 0.05; **P < 0.01; ***P < 0.001; ****P < 0.0001.

We further examined key insulin-signaling proteins by Western blotting (Figures 9B–D). Compared with insulin-stimulated control cells, TNF-alpha-treated adipocytes showed reduced phosphorylation of AKT and IRS1, consistent with impaired insulin signaling. Compared with the TNF-alpha plus insulin group, KO treatment significantly increased the p-AKT/AKT ratio (*P* < 0.01) and decreased the p-IRS1/IRS1 ratio (*P* < 0.05). FO and SL also produced changes in these markers, but their effects were less pronounced than those observed with KO. The higher p-AKT/AKT ratio in the KO group is consistent with enhanced downstream insulin signaling. Our cellular findings are consistent with previous reports indicating that krill oil and omega-3 fatty acids can modulate insulin-related signaling pathways, including the PI3K/AKT pathway (45). The phospholipid-bound form of omega-3 fatty acids may differ from neutral-lipid forms in absorption and cellular handling (46); however, the present study was not dose-matched for EPA and DHA. Therefore, the larger response observed with KO cannot be attributed solely to lipid chemical form. The response may also reflect the combined effects of phospholipid-bound omega-3 fatty acids and other KO constituents, including astaxanthin (47). Under the present experimental conditions, KO showed a greater effect than FO and SL on glucose uptake and AKT phosphorylation in TNF-alpha-treated 3T3-L1 adipocytes (48). These results provide cell-level support for the potential role of KO in modulating insulin signaling, while direct target engagement and *in vivo* efficacy require further investigation.

## 3 Materials and methods

### 3.1 Material and reagents

The KO, FO, and SL were all provided by Huache Biotechnology Co., Ltd. (Jiangsu, China). The lot numbers for the three oil samples were: KO (Batch No. HC-KO-20230801), FO (Batch No. HC-FO-20230815), and SL (Batch No. HC-SL-20230901). KO and FO were extracted by cold-press extraction from Euphausia superba and deep-sea fish (primarily anchovy), respectively, while SL was produced by enzymatic modification of soybean phospholipids. All samples were stored in sealed, light-protected containers at −20 °C from receipt until analysis. According to its test reports, KO contains 1.29 g/kg of eicosapentaenoic acid (EPA), 0.56 g/kg of docosahexaenoic acid (DHA), 4.19 g/kg of total phospholipids, and 2.82 mg/kg of astaxanthin; FO contains EPA ≥ 1.80 g/kg, DHA ≥ 1.20 g/kg, and total ω-3 fatty acids ≥ 3.50 g/kg; SL has an acetone-insoluble matter (an indicator of phospholipid purity) of 95.8% and an acid value of 30.4 mg KOH/g. All samples were stored in sealed and light-protected containers at minus 20 °C until use. LC-MS grade reagents such as methanol and acetonitrile were purchased from Merck; L-2-chlorophenylalanine (≥98%) was purchased from Shanghai Aladdin; formic acid was purchased from TCI; methyl tert-butyl ether (MTBE) was purchased from CNW Technologies. Mouse 3T3-L1 preadipocytes (American Type Culture Collection, Manassas, VA, USA) were cultured in Dulbecco’s modified Eagle’s medium (DMEM; Gibco, Carlsbad, CA, USA) supplemented with 10% newborn calf serum (NBCS; Gibco), 100 U/mL penicillin, and 0.1 μg/mL streptomycin (Gibco) at 37 °C in a humidified atmosphere containing 5% CO_2_.

### 3.2 Untargeted metabolomics (UHPLC-HRMS) analysis

#### 3.2.1 Metabolite extraction

First, 100 μL of the sample were put into a centrifuge tube. After adding 500 μL of extraction solvent with internal standard (methanol:acetonitrile = 1:1, v/v, final internal standard concentration 20 mg/L), it were vortex-mixed for 30 s. Then the ultrasonic extraction was performed in an ice-water bath for 10 min to promote complete release of metabolites. Subsequently, the sample was placed at minus 20 °C for 1 h to allow protein precipitation, followed by centrifugation at 12,000 rpm and 4 °C for 15 min. The 500 μL of supernatant was then carefully transferred into a new EP tube and dried in a vacuum concentrator. The dried residue was redissolved in 160 μL of reconstitution solution (acetonitrile:water = 1:1, v/v), and vortex-mixed for 30 s, and again ultrasonicated in an ice-water bath for 10 min, followed by centrifugation at 12,000 rpm and 4 °C for 15 min. Finally, 120 μL of the obtained supernatant were put into a 2 mL sample vial. Additionally, 10 μL from each sample were took and mixed with equal volumes to prepare quality control (QC) samples, which were used to assess instrument stability and data quality before instrumental analysis (45).

#### 3.2.2 Instrument conditions and data acquisition

Metabolomics analysis was performed using an ultra-high performance liquid chromatography–high resolution mass spectrometry (UHPLC–HRMS) system (consisting of a Thermo Dionex Ultimate 3000 UHPLC system coupled to an Orbitrap Exploris 480 high-resolution mass spectrometer). Chromatographic separation was achieved using a Waters ACQUITY UPLC HSS T3 column (2.1 × 100 mm, 1.8 μm). The same mobile phase system was used for both positive and negative ion modes: mobile phase A was 0.1% aqueous formic acid solution, mobile phase B was 0.1% formic acid acetonitrile solution, and the injection volume was 1 μL.

Mass spectrometry detection was performed using an electrospray ion source (ESI) in the data-dependent acquisition (DDA) mode to obtain secondary MS/MS fragment information, with a scan range of m/z 67–1000. The ion source parameters were set as follows: spray voltage at 3.5 kV (positive ion mode) or -2.5kV (negative ion mode), sheath gas flow rate at 50arb, auxiliary gas flow rate at 10 arb, sweep gas flow rate at 1arb, ion transfer tube temperature at 325 °C, and vaporizer temperature at 350 °C (36).

As for the data, the acquired raw data (.raw) files were imported into the CD (Compound Discoverer) software for simple filtering of parameters such as retention time and mass-to-charge ratio (m/z). Subsequently, peak alignment was carried out across different samples based on a retention time deviation of 0.2 min and a mass deviation of 5ppm to improve identification accuracy. Then, peak extraction was performed according to the following settings: mass deviation of 5ppm, signal intensity deviation of 30%, signal-to-noise ratio of 3, minimum signal intensity of 100,000, and adduct ion information. Meanwhile, peak areas were quantified, and target ions were consolidated. Molecular formulas were then predicted based on molecular ion peaks and fragment ions and were compared against the mzCloud, MZvault, and Chemspider databases. Finally, the identification and quantification results of the data were obtained.

### 3.3 Untargeted lipidomics (UPLC-QTOF-MS) analysis

#### 3.3.1 Lipids extraction

The oil sample was added to 200 μL of ultrapure water, followed by the addition of 500 μL of the extraction solvent (methyl tert-butyl ether:methanol = 5:1, v/v) and magnetic beads, and then processed in a grinder at 45 Hz for 12 min to fully disrupt and emulsify the sample. After vortex-mixing for 30 s, the mixture was ultrasonicated in an ice-water bath for 15 min, and stood at -40 °C for 1h to enhance phase separation efficiency, followed by centrifugation at 13,000 rpm and 4 °C for 15 min. Then, 300 μL of the supernatant was transferred to an EP tube and dried in a vacuum concentrator to remove the organic solvent. The obtained dried extract was reconstituted with 200 μL of dichloromethane:methanol (1:1, v/v), vortex-mixed for 30 s, and ultrasonicated in an ice-water bath for 10mins. After centrifugation at 12,000 rpm and 4 °C for 15 min, 140 μL of the supernatant was transferred into a 2 mL injection vial for analysis. To monitor instrument stability and data quality, 10 μL of each sample was mixed to prepare a quality control (QC) sample, which was analyzed simultaneously with the test samples (46).

#### 3.3.2 Instrument conditions and data acquisition

Untargeted lipidomics analysis was performed using an ultra-high performance liquid chromatography–high resolution mass spectrometry (UPLC–HRMS) system (consisting of a Waters ACQUITY I-Class PLUS UPLC coupled to a Waters Xevo G2-XS QTof high-resolution mass spectrometer). Chromatographic separation was achieved using an ACQUITY UPLC CSH C18 column (1.7 μm, 2.1 × 100 mm, Waters). The same mobile phase system was used for both positive and negative ion modes: mobile phase A was 60% aqueous acetonitrile solution (containing 10mM ammonium acetate and 0.1% formic acid), and mobile phase B was 90% isopropanol/acetonitrile solution (containing 10 mM ammonium acetate and 0.1% formic acid); the injection volume was 5 μL, and the column temperature was maintained at 55 °C. Chromatographic separation was performed using the following gradient program: 0–2 min, 60% B; 2–15 min, 60–90% B; 15–20 min, 90–100% B; 20–22 min, 100% B; 22–22.1 min, 100–60% B; 22.1–25 min, 60% B, with a flow rate of 0.4 mL/min. The mass spectrometer was operated in full-scan mode with a mass resolution of 40,000 FWHM at m/z 200 for MS scans and 20,000 FWHM for MS/MS scans. For data normalization and quality control, deuterated internal standards (Avanti Polar Lipids, Alabaster, AL, USA) were spiked into each sample prior to extraction, including PC(14:0/14:0)-d4, PE(14:0/14:0)-d4, and ceramide (d18:1/12:0)-d7. A pooled quality control (QC) sample was prepared by mixing equal aliquots of all samples and injected periodically throughout the analytical run to monitor instrument performance and signal stability, following the same procedures as described for metabolomics analysis (20).

Under the control of MassLynx V4.2 software (Waters), the mass spectrometer acquired primary MS and secondary MS/MS data in MSE mode, simultaneously collecting low-collision energy and high-collision energy data in each scan cycle. The low collision energy was set to 2V, and the high collision energy was set from 10 to 40V, with a scan rate of 0.2 s/scan. The ESI ion source parameters were set as follows: capillary voltage at +2500V (positive ion mode) or -2000V (negative ion mode), cone voltage at 30V, ion source temperature at 120 °C, desolvation gas temperature at 550 °C, backflush gas flow rate at 50 L/h, desolvation gas flow rate at 900 L/h, and mass-to-charge ratio scan range at m/z 50–2000. The raw data were acquired by MassLynx V4.2 and then imported to Progenesis QI software for peak extraction, peak alignment, and normalization. Metabolite identification was performed based on online databases and an in-house database, combined with theoretical fragment information for structural confirmation (24).

### 3.4 Network pharmacology and molecular docking

#### 3.4.1 Targets screening of krill oil components

The targets of the chemical components in the krill oil were retrieved using the SwissTargetPrediction and SEA databases. After the targets with a probability value of zero were removed and the duplicates were deleted, the final obtained targets were defined as the potential action targets of the selected KO-associated component annotations.

#### 3.4.2 Targets screening in insulin resistance and metabolic disorders

The targets related to insulin resistance and metabolic disorders were collected from the GeneCards^1^, OMIM^2^, and TTD^3^ databases using the following search keywords: “insulin resistance”, “type 2 diabetes mellitus”, “metabolic syndrome”, “obesity”, “glucose metabolism disorder”, and “lipid metabolism disorder” (28). After removing duplicates, a Venn diagram was used to obtain the intersection of krill oil component targets and disease targets, which were considered as the potential key targets for krill oil intervention in insulin resistance and metabolic disorders. Selection of representative components and target screening: Candidate components from krill oil were selected based on four strict criteria: (1) significant up-regulation in KO compared to both FO and SL (VIP > 1, | log_2_FC| > 1, *p* < 0.05); (2) representation of major differential lipid classes (e.g., omega-3 PUFAs and diacylglycerols); (3) unique functional presence in KO (i.e., astaxanthin); and (4) structural availability for target prediction. Consequently, 9 core differential components and 1 unique component were finalized. The targets of these selected components were then retrieved using the SwissTargetPrediction and SEA databases.

#### 3.4.3 “Component-target-illness” network establishment

A correspondence relationship between krill oil components and disease targets was established. The network was constructed and subjected to topological analysis using Cytoscape 3.8.2 software. Degree, betweenness centrality, and closeness centrality were calculated to screen for core components.

#### 3.4.4 Protein interaction network establishment and core targets screening

The above intersection targets were submitted to the STRING database^4^, with the species set to “Homo sapiens” and the remaining parameters kept as default. The obtained PPI network was exported in TSV format and further analyzed using Cytoscape 3.8.2. The targets ranked in the top 10 based on degree, betweenness centrality, and closeness centrality were defined as core targets.

#### 3.4.5 GO and KEGG enrichment analysis

GO functional enrichment analysis and KEGG pathway enrichment analysis were performed using the clusterProfiler package in R. Enrichment analysis was conducted based on the target gene sets or differential metabolite annotations obtained in this study. Pathways and GO terms with *P* < 0.05 were considered statistically significant. The top five GO terms from the biological process, cellular component, and molecular function categories and the top 10 KEGG pathways were selected for visualization. The enrichment figures were generated using the standardized output format of the sequencing-data analysis platform.

#### 3.4.6 Molecular docking verification

Molecular docking was performed using AutoDock Vina (version 1.2.0). The 3D structures of the four selected components, namely DAG, DHA, EPA, and stearidonic acid, were retrieved from PubChem and converted to PDB format using Open Babel (3.1.1). Receptor PDB files (AKT1: 4GV1; SRC: 2SRC; PPARG: 3DZY; IL6: 1ALU; STAT3: 6NJS) were downloaded from the RCSB Protein Data Bank. Receptor preparation (co-crystallized ligand and water removal, hydrogen addition, and Gasteiger charge assignment) was performed using PyMOL 2.5 and AutoDock Tools 1.5.7. The grid box was set to 60 × 60 × 60 Å^3^, with target-specific center coordinates specified for AKT1, SRC, PPARG, IL6, and STAT3. Docking exhaustiveness was set to 8, with 20 binding modes. The docking score of the lowest-scoring pose was recorded for each component-target pair. Docking results were interpreted as predictions for candidate prioritization, not as evidence of direct binding.

### 3.5 Cell culture and *in vitro* functional analysis

#### 3.5.1 Cell culture and adipocyte differentiation

Adipocyte differentiation was induced using a standard hormonal cocktail. Two days after reaching confluence, cells were designated as day 0, and the culture medium was replaced with differentiation medium consisting of DMEM supplemented with 10% fetal bovine serum (FBS), 0.5 mM 3-isobutyl-1-methylxanthine, 0.25 microM dexamethasone, and 1 microgram/mL insulin. After 72 h, the medium was replaced with DMEM containing 10% FBS and 1 microgram/mL insulin, and the cells were cultured for an additional 72 h. From day 6 onward, the cells were maintained in DMEM supplemented with 10% FBS, with the medium replaced every 2 days. Fully differentiated adipocytes were obtained on days 10–12 and were identified by the presence of abundant intracellular lipid droplets under phase-contrast microscopy.

#### 3.5.2 Oil treatment and induction of cellular insulin resistance

KO, FO, and SL were dissolved in DMSO to prepare 100 mg/mL stock solutions and diluted in serum-free DMEM to a final concentration of 100 micrograms/mL. The treatments were not matched for EPA or DHA content; therefore, the cellular comparisons represent the effects of the intact oil formulations rather than an equal-dose comparison of EPA/DHA chemical forms. The final DMSO concentration did not exceed 0.1% (v/v), and vehicle controls received an equivalent amount of DMSO. Mature 3T3-L1 adipocytes were seeded in 12-well plates at 1 × 10^5^ cells per well. To establish the insulin-resistance model, cells were treated with recombinant mouse TNF-alpha (10 ng/mL) for 24 h. For the intervention experiments, cells were pretreated with KO, FO, or SL (100 micrograms/mL) for 24 h and subsequently co-treated with TNF-alpha (10 ng/mL) and the corresponding oil for an additional 24 h. Rosiglitazone (20 microM) was used as a positive control where indicated.

#### 3.5.3 Glucose uptake assay

After treatment, cells were washed twice and equilibrated in KRPH buffer for 4 h. Cells were then stimulated with insulin (100 nM) for 30 min, followed by incubation with 2-NBDG (100 microM) for an additional 30 min at 37 °C in the dark. After three washes with ice-cold PBS, cells were lysed in 0.1 M potassium phosphate buffer (pH 10) containing 0.1% Triton X-100. Fluorescence was measured at excitation and emission wavelengths of 485 and 535 nm, respectively, using a BioTek Synergy H1 microplate reader. The fluorescence values were normalized to total protein content determined using the BCA assay.

#### 3.5.4 Western blot analysis

Following treatment, cells in the insulin-stimulated groups were exposed to insulin (100 nM) for 30 min before protein extraction. Cells were lysed in RIPA buffer containing protease and phosphatase inhibitors, and equal amounts of protein (30 micrograms per lane) were separated by 10% SDS-PAGE and transferred onto PVDF membranes. After blocking with 5% BSA, membranes were incubated overnight at 4 °C with antibodies against phospho-AKT (Ser473), total AKT, phospho-IRS1 (Ser307), total IRS1. Membranes were then incubated with the corresponding HRP-conjugated secondary antibodies, and protein bands were visualized using enhanced chemiluminescence. Band intensities were quantified using ImageJ. The p-AKT (Ser473)/AKT and p-IRS1 (Ser307)/IRS1 ratios were calculated and expressed relative to the control group.

### 3.6 Statistical analysis

For cell-based experiments, data are expressed as mean ± SD from at least three independent replicates. Group comparisons were performed using one-way ANOVA with Tukey’s *post hoc*-test for multiple groups, or unpaired two-tailed Student’s *t*-test for two groups. *P* < 0.05 was considered statistically significant. GraphPad Prism (version 8.0) was used for all cell-based statistical analyses. For untargeted metabolomics and lipidomics, all oil samples were analyzed with (*n* = 6) independent biological replicates per group. Data from the two platforms were processed and analyzed separately. PCA and OPLS-DA were performed to assess group separation. Differential features were screened using VIP > 1, | log_2_FC| > 1, and *P* < 0.05. Feature counts from metabolomics and lipidomics were reported separately and were not combined.

## 4 Conclusions

This study compared the molecular profiles of krill oil (KO), deep-sea fish oil (FO), and soybean lecithin (SL) using untargeted metabolomics, untargeted lipidomics, and network pharmacology. KO showed a distinct lipid composition characterized mainly by phospholipid-associated omega-3 fatty acids, cardiolipins, and signaling-related phospholipids, whereas FO and SL displayed different lipid profiles. Network pharmacology and molecular docking were used to prioritize candidate component-target relationships and generate mechanistic hypotheses; these computational results do not establish direct binding or target engagement. In a TNF-alpha-induced insulin-resistant 3T3-L1 adipocyte model, KO increased insulin-stimulated glucose uptake and the p-AKT/AKT ratio under the tested conditions, with stronger effects than FO and SL in the corresponding cellular comparisons. These findings provide cell-level evidence that KO may modulate insulin-related signaling, but they do not establish *in vivo* efficacy or prove that specific individual components are directly responsible for the observed effects. Further studies using EPA/DHA-matched formulations, targeted chemical validation, direct target-engagement assays (incorporating established positive controls), and animal models are needed to clarify the component-specific mechanisms and translational potential of KO.

## Data Availability

The data analyzed in this study were obtained in collaboration with Biomarker Technologies Corporation Co., Ltd. The following licenses/restrictions apply: proprietary database-related information, protection of commercial interests, and database security. The complete raw datasets cannot be publicly deposited. Requests to access the shareable, de-identified, or processed datasets should be directed to HL (ansishc@163.com).

## Footnotes

1
https://www.genecards.org/

2
https://www.omim.org/

3
https://db.idrblab.net/ttd/

4
https://string-db.org/
