# Supplementary material for: Multi-omics profiling and cellular validation reveal distinct lipid networks of krill oil and its predicted mechanisms against insulin resistance
Source: Front Nutr. 2026 Sep 23;13:1925480. doi: 10.3389/fnut.2026.1925480 (PMC13642128; doi:10.3389/fnut.2026.1925480)
Supplement: Supplementary file 1 [file Supplementary_file_1.docx]

Supplementary Material

## 1.1 Supplementary Figures

A

B

C

D

**Fig. S1.** KEGG enrichment network analysis of differential metabolites was performed for KO vs FO in positive ion mode (A) and negative ion mode (B), and for KO vs SL in positive ion mode (C) and negative ion mode (D).
